# Disruptions to shared mental models from poor quality of service in collaborative virtual environments

**DOI:** 10.1038/s41598-021-02567-7

**Published:** 2021-12-07

**Authors:** Nicholas Milef, Adam Ryason, Di Qi, Samuel O. Alfred, Cullen D. Jackson, Suvranu De

**Affiliations:** 1 Department of Computer Science and Engineering, Texas A&M University, College Station, TX 77843 USA; 2 Center for Modeling, Simulation, and Imaging in Medicine, Rensselaer Polytechnic Institute, Troy, 12180 USA; 3 Beth Israel Deaconess Medical Center, Boston, MA 02215 USA; 4 Harvard Medical School, Boston, MA 02215 USA

**Keywords:** Electrical and electronic engineering, Computer science, Information technology

## Abstract

Collaborative virtual environments are being used in various applications ranging from online games to complex team training scenarios. The key to the success of such environments is the ability of the participants to form a shared mental model of the collaborative task being performed. Poor quality of service can deteriorate user performance and quality of experience, leading to a disruption of this mental model. While the effects of quality of service have been analyzed for traditional desktop environments, these effects remain unclear in collaborative virtual environments during user-to-user interactions. Here, we analyze the role of latency and packet bursts, two common problems in collaborative applications, on both simulator perception and actual task performance in a collaborative fire-fighting simulator. This exploratory study indicates that large latencies have a significant (p < 0.05) impact on the quality of experience, but not task performance. In contrast, packet bursts have a much larger impact on both the quality of experience and performance. Additionally, the network role, such as whether a user is a client or server, showed a significant (p < 0.05) impact on task performance in conditions impaired by packet bursts.

## Introduction

With the rise in popularity of multiplayer gaming and consumer head-mounted display (HMD) systems, multi-user virtual reality (VR) applications have become ubiquitous. VR collaborative virtual environments (CVEs) are virtual reality applications where users can interact with one another to accomplish a larger goal. VR CVEs enable the creation of a wide range of entertainment and team-training applications^1–5^. For example, CVEs in gaming can promote teamwork through shared objectives, encouraging participants to share resources and take on specific roles. Other collaborative applications include team training, which is a critical component of many professions such as emergency response and surgical teams. To accomplish these collaborative tasks, it is critical for participants to construct a shared mental model (SMM)^6–9^. In psychology, an SMM is a shared understanding of the environment and tasks among a team of participants, and this shared understanding is often critical for improving performance in collaborative tasks. However, this SMM can be disrupted by poor quality of service.

Computer networks that provide the backbone to CVEs are subject to unpredictable drops in performance. Network connection quality, also known as quality of service (QoS), can vary significantly due to underlying network infrastructure and network congestion. Poor QoS can deteriorate user performance and the quality of experience (QoE), the user’s perceptions of how the environment affects his/her performance, in networked applications. QoS is characterized by multiple factors that affect QoE: latency (time delay from sending to the reception of a packet)^10–18^, jitter (the variance of latency)^13,19^, and packet loss. Unstable internet connections and various sources of delay, such as infrastructure and application-level code, are common causes of poor QoS. Even as internet infrastructure and standards improve, mitigating the effects of latency remains a significant technical challenge. For example, both LTE round-trip times^20^ and Wi-Fi^21^ can add hundreds of milliseconds of latency to a networked application. Unfortunately, because of the real-time nature of video games and 3D interactive simulations, latency and jitter have been shown to have a substantial impact on task performance and QoE in these applications^10,11,22^. In developing countries, these conditions can be even worse, limiting the market penetration of collaborative VR applications and raising questions of fairness among participants with differing QoS^23^.

Collaborative tasks benefit from the construction of SMMs^7,9^. When the QoS worsens, each instance of the shared virtual world diverges. In turn, the SMM can deteriorate, making it more difficult for participants to complete tasks that require team work. SMMs are formed through team learning behaviors including construction, co-construction, and constructive conflict^9^. Participants engage in “construction” when they perform actions or make decisions such as tossing a fire extinguisher in our simulator. “Co-construction” involves other participants building upon construction behaviors, such as catching the extinguisher. “Constructive conflict” occurs when team members do not agree on the SMM, which can occur due to interruptions in network connectivity. In our study, we explore how different types of QoS disruptions can shift the team learning behavior.

While user performance and QoE as a result of QoS has been studied in competitive and collaborative games, as far as we are aware, there has not been a comprehensive analysis of the effects of both latency and packet bursts in HMD-based VR tasks requiring user-to-user collaborative interaction. We developed a multi-user simulator as a platform to collect data on the effect of various QoS metrics in VR CVEs. We then conducted a study to gather data on user behaviors as affected by these network conditions.

Through our user study, we provide answers to the following research questions in the context of collaborative VR environments with person-to-person interactions:

- What effects do high latencies have on both the perception and actual task performance of users?
- Does a user’s role (client/server) in the network have an impact on QoE or task performance?
- Can latency offset the effects of packet bursts?

By answering these questions, we can better understand user tolerances and preferences in VR CVEs. These answers provide insight into the potential directions that could be taken when designing networking architectures for collaborative applications. Optimal network designs can reduce the degradation of SMMs under poor network conditions, leading to better QoE and task performance.

## Methods

### Study design

To measure the effects of QoS on SMMs, we developed a VR simulator inspired by a real training scenario for extinguishing a fire in an operating room^24,25^. To assist in future analysis, we recorded the trajectory data of the extinguisher, hands, and head of each user. We also recorded fire extinguishing time and metadata, including who has authority over the fire extinguisher at a given time. We simulated different network conditions using software and conducted a user study to answer our research questions.

#### Task details

**Figure 1:**
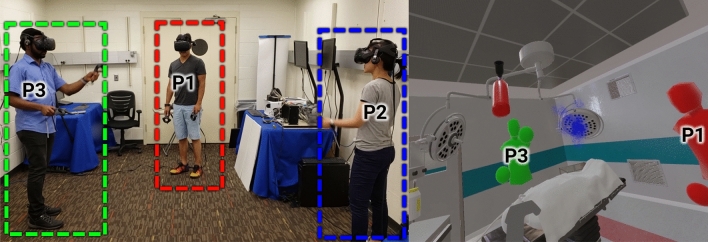
A group of users working through a trial of the multiplayer simulator at the network control conditions. Participants are outlined in their respective avatar color in the VR environment. P3 (green, left) is tossing a virtual fire extinguisher to P2 (blue, right) while P1 is viewing the interaction (middle, red). The VR rendering is from P2’s perspective, so P2 is not visible in the image on the right.

**Figure 2:**
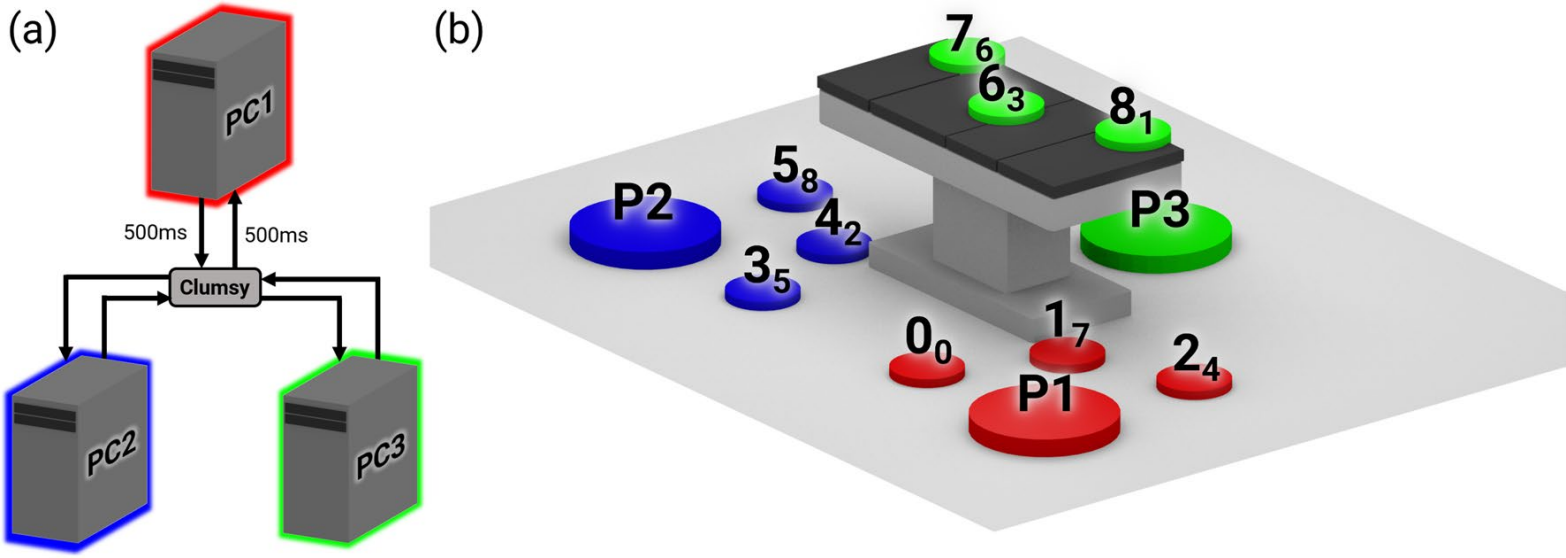
(**a**) The network topology for our system. PC1 is connected to both PC2 and PC3, but PC2 and PC3 are not connected to each other. In this example, 500 ms of latency is being applied using a software tool Clumsy. (**b**) The VR room layout for various user positions and a possible fire spawn order. The small circles represent the fire locations, and the larger circles represent the starting locations for each user. Each number is the fire location ID, and each subscript represents an example ordering of the fires (i.e., the order in this example is 084623715).

In our simulator, three participants work on a collaborative task in a virtual environment, and each participant is represented by an avatar (Fig. 1). Each participant’s avatar is a 3D model that is animated based on the participant’s hand and body positions, which we acquire in real-time from VR tracking hardware. Participants are tasked with putting out fires at predefined locations within their vicinity, as shown in Fig. 2b. Each participant is connected to a computer and assigned a position; the server PC (P1) and the client PCs (P2 and P3) are positioned in a triangle (Fig. 2b). In our scenario, the participants are assigned a color (P1red, P2blue, or P3green) and must extinguish three of their own fires, which are color-coded based on their position. Each participant can only extinguish respective fires. For each fire color, there exist three possible locations for each fire color located close to the corresponding participant. Since there is only one fire extinguisher in the CVE, these three players must physically exchange the extinguisher to one another. This is carried out by grasping, swinging, and then releasing the fire extinguisher towards a target to provide the object a trajectory. The receiving player must then grasp the fire extinguisher once it is within range before it falls to the ground or passes the receiving player.

Each fire spawns sequentially; once one fire is extinguished, another spawns until all nine fires are extinguished. A few rules dictate the fire spawning behavior: (1) the first fire is always the same (fire 0); (2) for every three fires, all three colors must be represented (i.e., fires 0, 1, and 2 will be different colors); (3) two sequential fires cannot have the same color. These constraints were created to ensure that the virtual fire extinguisher was tossed from one user to another after extinguishing each fire and to ensure that each participant was actively engaged throughout the trial. This also creates an environment in which the users need to understand how they need to coordinate to fulfill each others’ tasks and complete the shared objective of extinguishing all of the fires. This randomized fire placement was designed to minimize the ability of participants to learn the order of who would throw the extinguisher next and anticipate the next throw, which forces the users to maintain a high level of situational awareness.

#### Network presets

**Figure 3:**
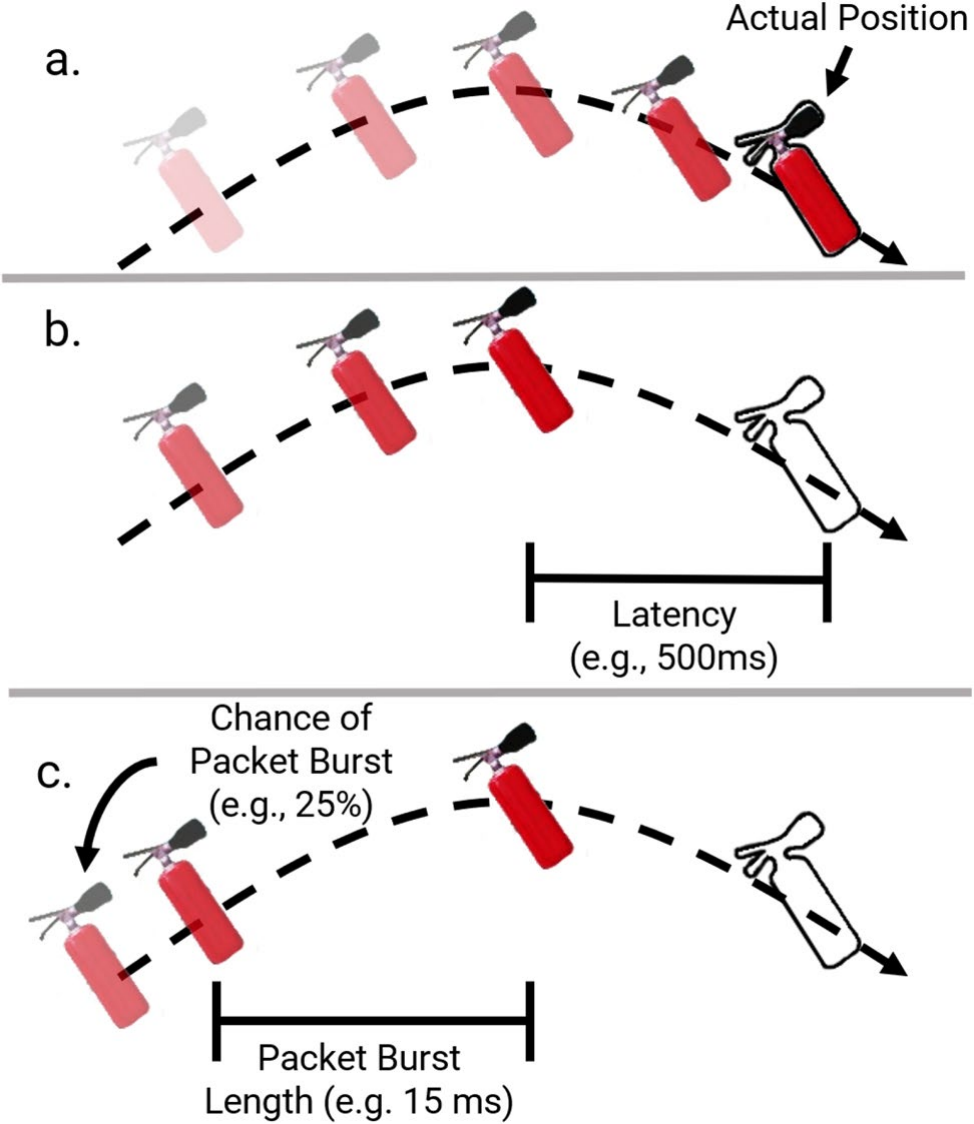
An illustration of the different preset behaviors. The outlined extinguisher represents the actual location (as defined by the authority of the extinguisher). The translucency corresponds to the recency of each position. Figures: (**a**) control (no latency or packet bursts, (**b**) latency but no packet bursts, and (**c**) latency and packet bursts.

For the user study, we tested five different conditions (Table 1). In these conditions, we varied three variables: latency, packet bursts, and packet burst chance (Fig. 3). We measure latency as the delay artificially added along one direction (i.e., not round-trip). However, this latency is still present in both directions; e.g., the round-trip latency of 1000 ms is 2000 ms. The latency presets take into account simulated latency and does not include internal application latency such as additional latency incurred by frame time, nor does it include latency incurred by the ethernet connection. We use 0 ms (baseline) as our baseline. The other latency values of 500 ms and 1000 ms are within the range of values tested in other latency studies^10,11^. Latency tolerance generally depends on the application type and associated mechanics. For example, Claypool and Claypool^10^ found that first-person avatar, third-person avatar, and omnipresent games have latency tolerances of 100 ms, 500 ms, and 1000 ms, respectively.

While some network conditions such as latency are well-defined and straightforward to emulate, other conditions such as jitter are not. Jitter does not follow a uniformly random distribution across real-world networks and can be largely masked by packet reordering schemes; Qin et al. showed that packet loss that fails to model burst loss might not be perceivable by users^26^. In our studies, we evaluate the effect of these “packet bursts” (also known as jerkiness^27^). Our goal in evaluating packet bursts is to measure the effect of “lag,” a loosely defined term for the responsiveness of an application often used by gamers to quantify the QoS^28^. Unlike uniform jitter, the effects of packet bursts have not been extensively studied.

Packet bursts, which simulates a cumulative effect that could be caused by network issues such as packet coalescing, low update rates, and packet congestion, is defined by two values: the amount of time to block all incoming/outgoing packets and the chance of this blockage to occur for a given packet. Because the chance is applied per packet, more packets sent during a frame contributes to a higher chance of triggering a data throttle. In our simulation, the server sends out 15 packets, so there is a high chance of a trigger occurring each frame. However, because the packets are sent sequentially, an individual object may not be throttled each frame. Additionally, once the throttle time period ends, all blocked packets are sent. This closely simulates the hitches caused by TCP correction during packet loss^29^. Similar effects can also occur in UDP, such as through burst loss. Similar to latency, the values are along one direction.

We introduced two packet burst conditions to replicate the behavior of lag. We found through preliminary testing that uniformly distributed jitter poorly emulated real-world lag, particularly with packet order enforcement. Suzejevic et al. incorporate a QoS measure of “jerkiness” which also includes a periodic slowdown by overloading the system with randomly spawned processes to freeze the application^27^. We take a more systematic approach by introducing this freezing mechanism at the network level, and we determined parameters for packet bursts that appeared to best emulate lag through preliminary testing. Our questionnaire responses regarding lag confirmed that our packet bursts settings were a reasonable proxy for lag (Fig. 7).

**Table 1:** Network condition presets for the user study.

Preset	Latency (ms)	Data throttle (ms)	Chance (%)
1	0	0	0
2	500	0	0
3	1000	0	0
4	500	15	25
5	500	30	50

#### Participants

We recruited 20 participants (age mean = 22.15, SD = 2.70) for our study. Prior to the start of the study, each participant was asked to fill out a demographic questionnaire. None of the participants reported having prior motion sickness in VR. Eleven (55%) of the participants reported having prior VR experience. Eight (40%) of the participants regularly play video games each week.

#### Study procedure

The study protocol was approved by the Rensselaer Institutional Review Board (IRB). Signed informed consent was obtained from each of the participants in the study. All methods were performed in accordance with relevant guidelines and regulations. At the start of each study group, we introduced the participants to the simulator. We allowed each participant to learn the controls through a test trial without impaired network conditions. This practice trial was designed to mitigate the practice effect and to familiarize the participants with the controls and the virtual environment.

Three participants were selected to participate in five trials (with each network preset); each trial consisted of each user extinguishing their three fires for a total of 9 fires extinguished per trial. The presets were selected through block randomization to mitigate the order effect. After the five trials, the participants either changed positions or were replaced by a waiting participant. Each participant participated in 15 total trials at the three different positions. Each study group took no longer than 2 h.

At the start of each trial, the participants were asked to return to the starting circles on the ground corresponding with their starting locations. P1 always started the simulator and extinguished the first fire. After each trial, the three participants were asked to fill out a questionnaire about their perception of the network conditions; each participant completed 15 post-trial questionnaires in total.

### System design

We constructed our networked simulator using the Interactive Medical Simulation Toolkit (iMSTK) (https://www.imstk.org/). We utilized iMSTK’s Vulkan backend^30^ to render the VR environment efficiently. To simulate the rigid body dynamics, we used Nvidia’s PhysX framework and advanced the physics simulation at 60 Hz. Each computer in the networked simulator runs a redundant copy of the scene, so each is responsible for computing physics and animation.

### Simulator dynamics

Our VR scene simulates a virtual operating room. Our VR operating room setup included a rigid body attached to the fire extinguisher, and static rigid bodies for the walls, floor, and ceiling. Collisions are absent between the extinguisher and other objects in the operating room, such as the operating table and medicine cabinet, to prevent instabilities from occurring if the user releases the extinguisher within an object. The extinguisher’s position is affected by gravity, collision response from the 3D operating room, and hand movement when held by a participant. We used particle systems to represent the extinguisher foam and the fires around the operating room. All particle effects were simulated locally.

Each user has an instance of the simulator running on a desktop Windows 10 PC with an RTX 2070 GPU and an i7-6850k CPU. To visualize and interact with the simulator, our users each had an HTC Vive with a single controller. In our studies, the computers are connected through ethernet over a Local Area Network (LAN) and are in a single room, allowing all participants to share the same tracking area and thus use the same tracking hardware.

#### Network architecture

Our network design only uses the UDP protocol, and each computer sends update packets at a fixed frequency, which in our case was 30 Hz. UDP does not include functionality to ensure the redelivery of dropped packets. Thus, to ensure critical information such as simulation state changes and object authority are updated on the server, each computer sends packets for objects they control. However, in our study, it is unlikely for a significant number of packets to be lost because the computers were connected through ethernet in the same room. In the fire extinguisher scenario, all simulation states are tied to the user who possesses authority over the extinguisher. The state is then redundantly sent to the server until another user acquires the extinguisher.

While UDP lacks some useful features that TCP provides, it can more easily be customized with features implemented at the application layer. One of the most important features is the ability to broadcast packets, sending them to multiple network endpoints at a given time through a single port. Another benefit is the ability to ignore lost or out-of-order packets. Because our simulation environment operates in real-time, receiving packets in a timely manner is a priority. Out-of-date packets are simply discarded if newer packets have already arrived. We use a redundant state design, so dropped packets are less of a concern; for example, if a user requests authority, that request is embedded in all future packets until authority is given to another user. With this approach, authority can still be updated even if the initial authority request packet is lost. On the other hand, TCP will resend lost packets and packets that are out-of-order to ensure correct sequencing of packets, which can lead to increased latency and grouping of packets that behave similarly to our packet bursts preset.

During each frame, the server sends 15 packets, each 512 bytes, to prevent fragmentation, to the clients. Each client will send less than 15 packets, but the exact number depends on the authority of the various networked objects. Each object is encapsulated within a single packet. Additionally, a ping packet is sent from each client to the server, and a ping packet is sent from the server to the clients. The ping packet contains information about the current state of the networking environment, such as local time and client ID number, and it is also used to initially connect the clients to the server.

We simulated the latency through a software tool called Clumsy (https://github.com/jagt/clumsy), which intercepts packets and buffers them to simulate various network conditions such as latency and packet bursts. We configured it to affect all inbound and outbound UDP traffic through the simulator’s port. Because both clients are not directly connected to one another, the networking preset values are effectively doubled when interacting between the two clients (Fig. 2a). For example, when the user on client 1 throws the extinguisher to the user on client 2 at network preset 1 (500 ms latency), the actual latency between the two clients is 1000 ms.

#### Authority model

Some approaches have attempted to mitigate the effect of poor QoS. Common techniques include using pre-recorded animations to mask the appearance of latency^31^ and correcting local state drifts^32^. An authority model can also mask poor QoS by changing control of various objects^33–35^. For example, if only one user operates an object at a given time, then that user should control the dynamics of the object for all other users until ownership is transferred. These ownership transfers can be made through heuristics^36^ or through logical transfer policies^29^. For our study, we built an authority system and evaluated it under both latency and packet bursts network impairments.

**Figure 4:**
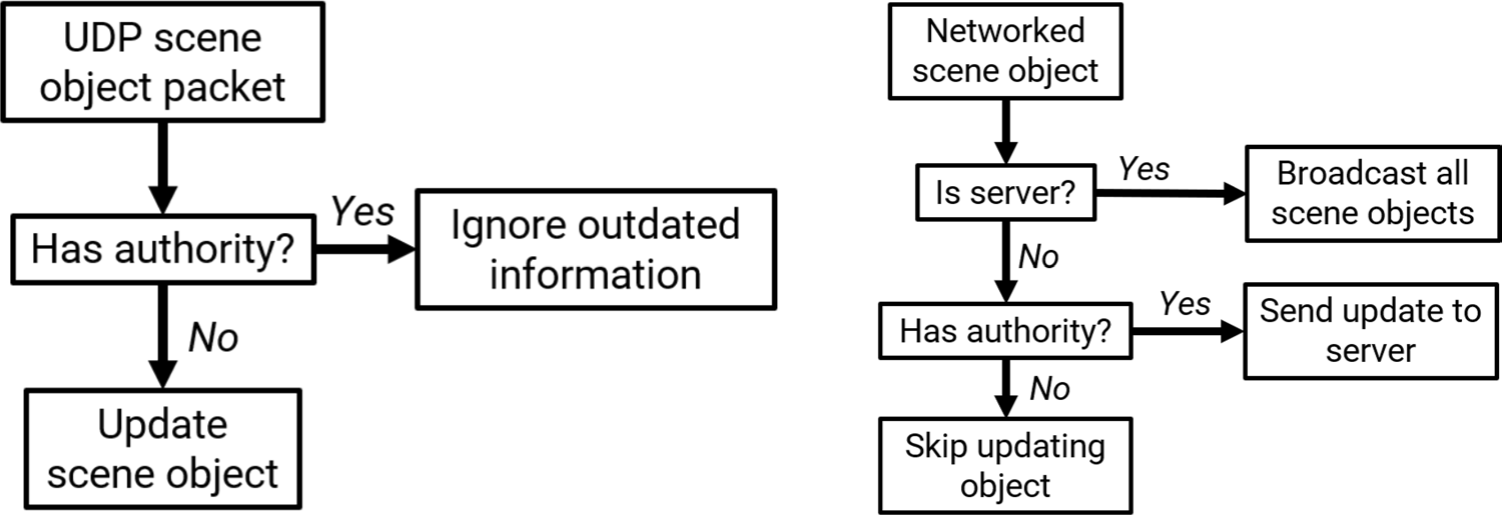
High-level authority model flow chart for a network node for (left) receiving and (right) sending packets.

We employed this authority model for determining ownership of an object across the network^29,37^ to reduce the effect of latency and prevent divergence with physics calculation (see Fig. 4). In general, it is more desirable to minimize the user’s local latency, such as latency from input (e.g., a controller) to visualization, than the latency of other connected users^18^. An example where physics divergence could occur would be if one user picks up the extinguisher. The user holding the extinguisher should have full control of its dynamics. However, without an authority model, other connected clients would apply gravity to the extinguisher between network updates, leading to an incorrect state of the extinguisher.

When one user possesses authority over an object, that user’s computer will be in charge of calculating its physics and sending the new positions and velocity to the server, which then broadcasts the whole simulator’s state to every client. The benefit of an authority model is that it can significantly simplify the synchronization of physics across multiple computers. In our case, since the extinguisher is the only dynamic rigid body, the authority models solve the synchronization problem of diverging physics states. Ryan et al. propose a similar idea with respect to user-object proximity in which objects close to remote users are updated with the same latency to hide discontinuities^38^.

A single computer can either serve the role of a server or a client PC. In the server role, the computer is tasked with listening to all connected clients and broadcasting the appropriate changes. For example, if a client has authority over an object, then that object will be sent to the server, which will broadcast this object to all other clients. To avoid ownership conflicts, the last user to request authority will be granted authority by the server. However, any user who requests authority will acquire authority locally until new updates from the server confirm otherwise. This allows users to pick up and use objects without any latency. Users can request authority of the extinguisher by grabbing the object, which in our simulator involved pressing the trackpad on the HTC Vive controller. To drop the object, the user releases the trackpad. To activate the extinguisher, the user simply pulls the trigger.

In our simulator, the owner of the extinguisher also controlled the simulator state, which included various pieces of essential information such as the current fire and how long the current owner has been trying to put out the fire. The position and focal point of the particle system were also transferred so that they could be rendered on each client without transmitting the positions of each particle.

## Results

### Performance results

We measured users’ task performance under the different network conditions using trial completion time and number of errors. The perceptual differences (QoE), as experienced by the users, were measured using a questionnaire that was provided at the end of each trial. Each trial consisted of participants P1 (red), P2 (blue), and P3 (green), where P1 served as the central server role. We study the latency presets (0 ms, 500 ms, and 1000 ms) and the packet burst presets (500 ms + 15 ms@25% and 500 ms + 30 ms@50%).

#### Number of drops (errors)

Errors were measured by the number of drops, or missed catches, made by the receiver of the fire extinguisher. We found that catching the extinguisher became very difficult in poor network conditions due to the unpredictability of delivered packets. Measuring the number of drops presented a few challenges. First, the trajectory of the extinguisher diverged among different participants. For example, if P1 threw the extinguisher, then P1 controlled the physics calculations such as gravity or bouncing off a wall. With latency present, the extinguisher would appear to have been dropped by the receiving client from P1’s perspective. These differences in user’s perspectives make it difficult for teammates to effectively coordinate their behaviors and understand the performance of the team. Second, in some cases, particularly those with severe packet bursts, when and if the extinguisher made contact with the ground became difficult to judge due to the application’s rejection of outdated packets; rejected packets resulted in missing positions along the extinguisher’s trajectory.

To accurately count the number of drops, we created a replay system to allow us to visualize synchronized trajectories; the trajectory that was used was the receiving user’s trajectory to ensure that we were viewing what the receiver was viewing. Additionally, we only counted a successful catch if the receiving user retained control. If the user briefly caught the extinguisher but then dropped it almost immediately, it was considered a drop. Finally, if a user mishandled the extinguisher during the throw and dropped it but later picked it up and reattempted the throw, the throw did not count as a drop.

**Figure 5:**
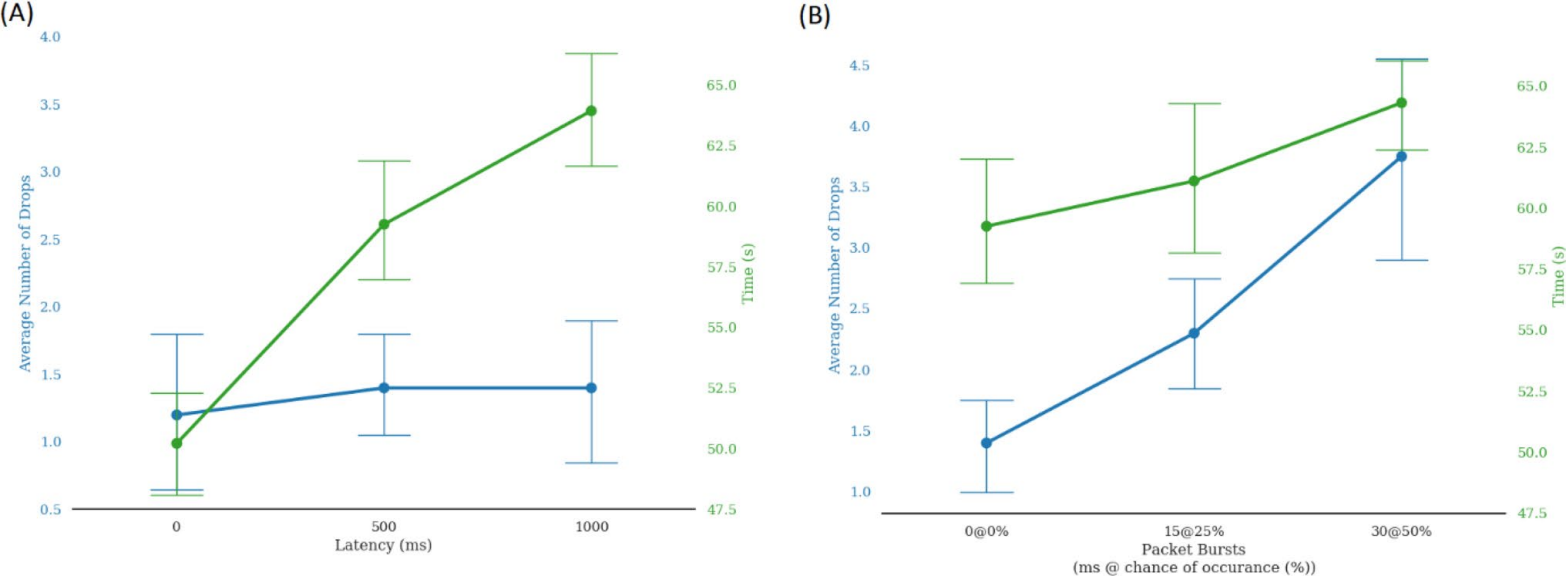
(**a**) The effect of latency on the number of errors (blue) and the completion time (green). (**b**) The effect of packet bursts on the number of errors (blue) and completion time(green).

To analyze the main effect of latency, we conducted a one-way repeated-measures ANOVA comparing the performance between latencies of 0 ms, 500 ms, and 1000 ms (Fig. 5a). Our results show that the drop rate was not significantly affected by the amount of latency, F(2,38) = 0.23, p = 0.796, $\eta$2 = 0.02. Mauchly’s Test of Sphericity indicated that the assumption of sphericity had not been violated, p > 0.05, and no correcting term was needed.

While latency did not have a significant impact, our results show that the drop rate was significantly affected by the degree of packet bursts. To analyze the effect of packet bursts, we compared the 500 ms, 500 ms + 15 ms@25%, and 500 + 30 ms@50% tests (Fig. 5b). There was a significant effect of packet bursts on drop rate within-subjects, F(2,38) = 18.496, p < 0.0005, and a large effect size of $\eta$2 = 0.9. Looking at the within subject contrasts, there was a significant effect and large effect size between 500 ms and 500 ms+15ms@25%, F(1,19) = 9.7, p = 0.006, $\eta$2 = 0.83, as well as between 500 ms + 15 ms@25% and 500 ms + 30 ms@50%, F(1,19) = 12.3, p < 0.0005, $\eta$2 = 0.88. Mauchly’s Test of Sphericity indicated that the assumption of sphericity had not been violated, p = 0.113, and no correcting term was needed.

**Figure 6:**
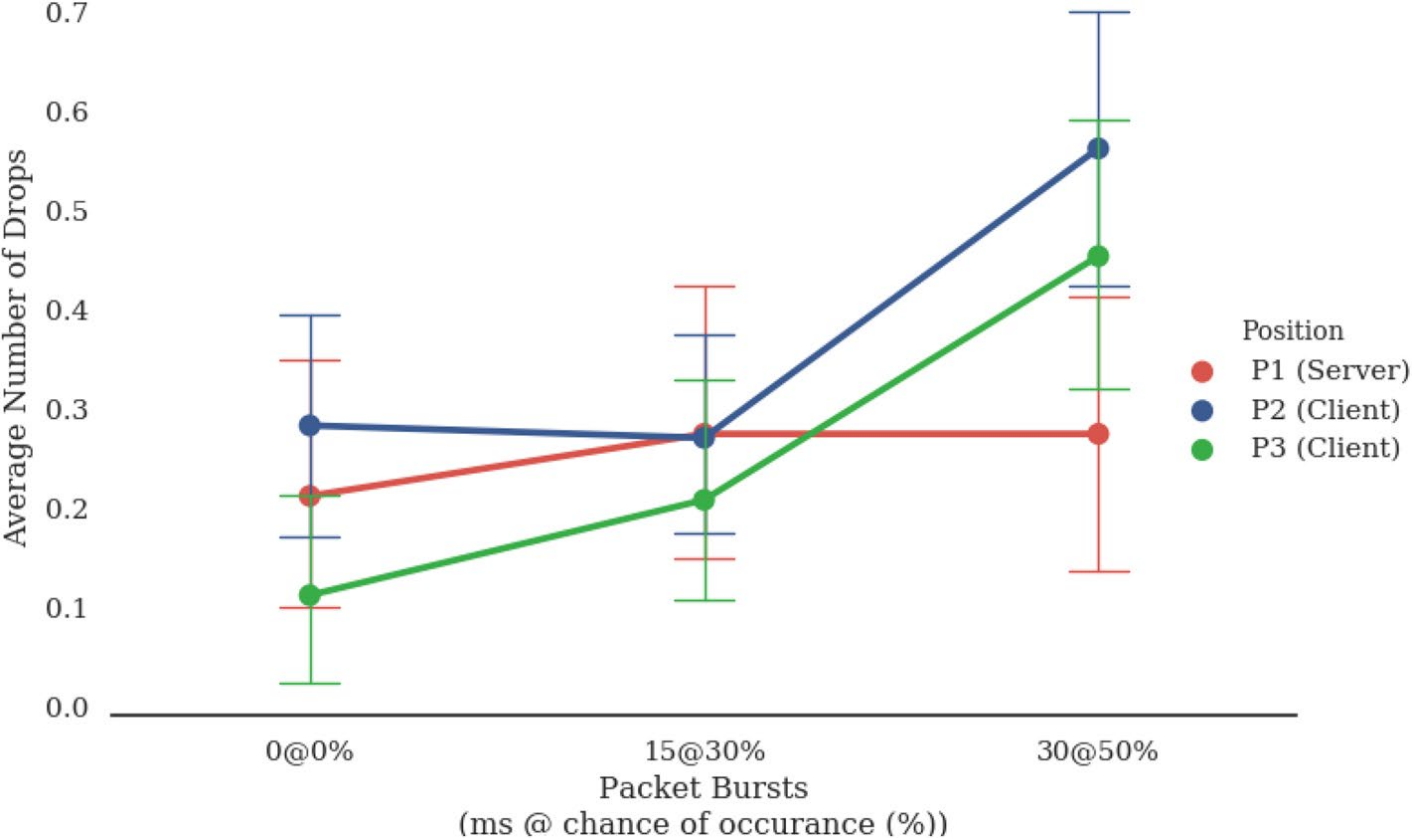
Frequency of drops (errors) by network role (position). Users whose computers functioned as the server performed better than users with client computers with the most packet bursts.

Our results showed a significant combined effect of position and packet bursts (Fig. 6). Position P1 (server) did not see a significant difference between the three throttling conditions while the other two positions (clients) did. Both P2 and P3 saw significant effects when contrasted with P1, between 500 ms + 15 ms@25% and 500 ms + 30 ms@50% for P2, F(1,19) = 4.77, p = 0.042 and between 500 ms and 500 ms + 30 ms@50% for P3, F(1,19) = 4.53, p = 0.047. Both P2 and P3 saw large effect sizes as well, $\eta$2=0.55 and $\eta$2 = 0.52, respectively.

#### Completion time

Completion time can serve as a valuable metric for task completion for the entire team in a collaborative environment. We measured the completion time for each trial by using the time the last of the nine fires was extinguished, as reported by P1. Higher latencies required a longer completion time partly due to the reaction delay to the other users, so latency has a direct effect on team task completion time irrespective of each user’s actual performance. It is important to take this into account when evaluating performance because a longer time-to-completion may not mean poorer per-user task performance depending on the context.

Completion time is much easier to compare in the packet burst scenarios. The time of completion increased when compared to the baseline latency preset (500 ms). The mean time for completing a single trial was 59.77 s, with a standard deviation of 7.57s. Between the three latency presets (0 ms, 500 ms, 1000 ms), we found the effect of latency on completion time to be significant (p = 0.001) through a one-way repeated ANOVA (Fig. 5a). Between the three packet burst presets, our data showed that the effect of packet coalescing was significant on completion time (p < 0.0005) (Fig. 5b). We performed within-subjects contrasts and saw a significant effect of completion time between 500 ms and 500 ms + 30 ms@50% but not between 500 ms and 500 ms + 15 ms@25% (p = 0.145). Mauchly’s Test of Sphericity indicated that the assumption of sphericity had not been violated for the comparison of completion time, p > 0.05, and no correcting term was needed.

### Quality of experience results

We measured the quality of experience (QoE) as perceived by the users through a questionnaire about perceived network performance that was completed by each user at the end of each trial. We asked users to rate five subjective questions on a Likert scale (1 lowest − 5 highest); Table 2 shows the questions from this survey.

**Table 2:** Reported questions from post-questionnaire.

Question	Scale
How did you perceive the network connectivity performance?	1-Poor
	5-Excellent
How noticeable was the network lag during the simulation?	1-Very noticeable/unplayable
	5-Unnoticeable
How responsive was your collaborator’s avatar movement?	1-Jerky movement
	5-Smooth movement
To what degree did the networking affect your performance?	1-Unable to complete the task
	5-No impact
How easy was it to interact with your collaborators?	1-Very difficult
	5-Very easy

#### Group perception

**Figure 7:**
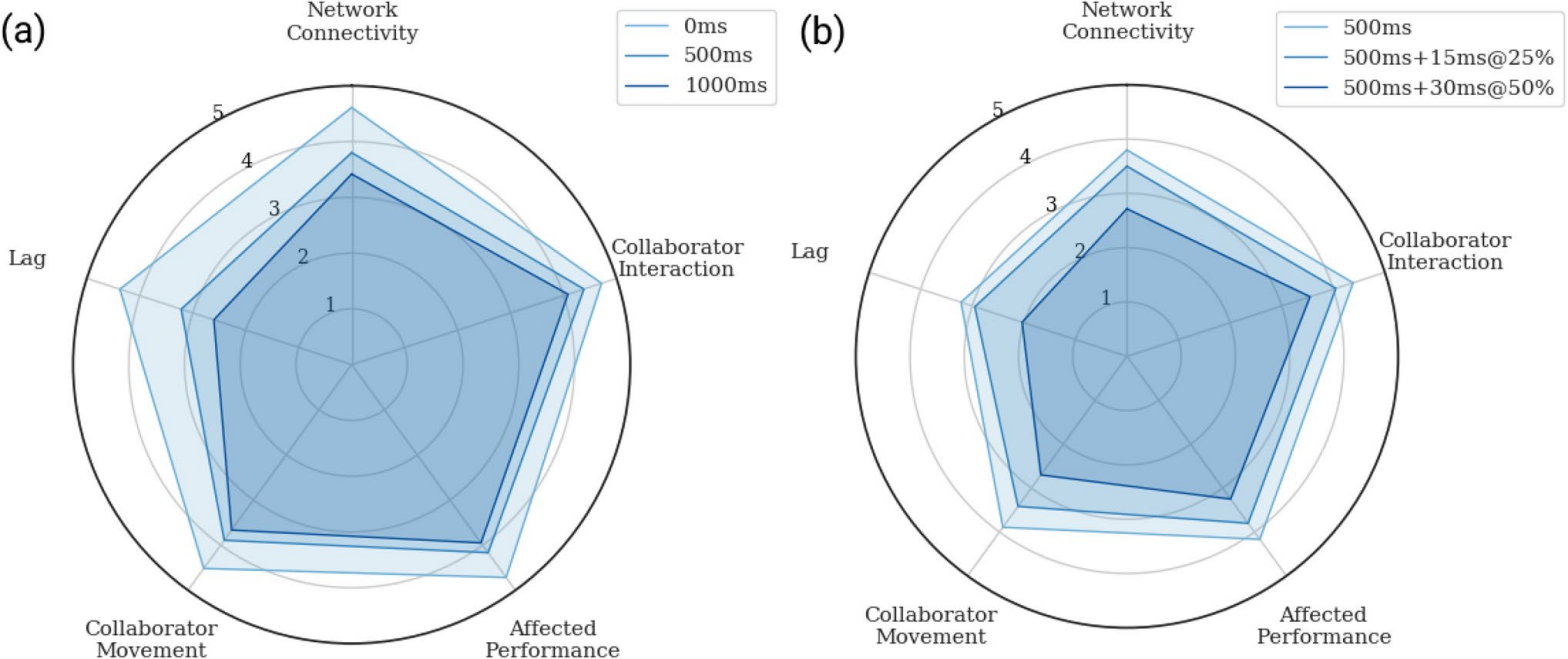
Questionnaire responses. (**a**) Questionnaire responses across different latencies. (**b**) Questionnaire responses across different packet burst levels.

Each group evaluated the simulator worse in accordance with a decline in QoS. Both latency and packet bursts had a significant effect (p < 0.0005) on the response for all five subjective questions. Increasing the latency had large effect sizes, with a mean of $\eta$2 = 0.92, on QoE, while packet bursts had similar effect sizes, with a mean of 2 = 0.94 on QoE. However, each aspect of networking was not perceived the same. In particular, the perception of collaborator interaction was not as influenced by worsened QoS, especially with regards to latency where an effect size of $\eta$2 = 0.77 was observed. Mauchly’s Test of Sphericity indicated that the assumption of sphericity had not been violated for any of the questions, p>.05, and no correcting term was needed. A radar plot of the perception under the given network conditions is presented for latency (Fig. 7a) and packet bursts (Fig. 7b).

#### Perceptual results with respect to network role

Although there was a significant change in perception for different network presets, we did not observe a significant effect on questionnaire responses due to network role (p > 0.05). Users appeared to give consistent feedback, despite their difference in positioning.

## Discussion

### Analysis of group performance and perception

Through our study, we were able to determine how latency and packet bursts affected a user’s perception and behavior. Latency had little impact on the average number of errors made by each user. However, increased latency led to worse perception of network conditions, although not to the same extent as the packet burst presets. Lastly, latency increased completion time, although this is largely due to the added delay in network communication. While the exact simulation differs for each user, the overall movement behavior and intent was preserved, resulting in the preservation of the SMM through refining their approximate interpretation of the virtual environment (i.e., co-construction).

On the other hand, the packet burst conditions had a larger impact on both QoE and actual task performance. The added completion time was likely due to error correction methods (i.e., picking up the dropped fire extinguisher). Additionally, the drop rates and QoE were much worse as compared to the baseline condition of 500 ms. Packet bursts will cause the trajectory of remotely-controlled objects to appear choppy. While the fire extinguisher is moving through the air, the flight path of the extinguisher effectively loses some of its frame updates, which makes predicting the current position difficult. An increase in the chance of packet bursts has the effect of increasing the uncertainty of where the extinguisher will be in the next update. These significant changes in simulation state lead to constructive conflict of the SMM. Although participants diverge significantly in their interpretation of the correct state, participants are able to adapt to these diverging conditions at the expense of task efficiency. This can have a critical effect on decision making in high-stakes, fast paced, team-based virtual environments such as surgery^39–41^, aviation^42–45^, and robotics^46,47^. In these situations, mistiming of information retrieval and situation assessment between team members can lead to improper responses.

While it would be best to minimize latency to below the values selected in our trial, adding latency can be applied rather liberally to stabilize the simulation^23^. If the packet bursts or related conditions (such as jitter and packet coalescing) were to occur, additional buffering and interpolation should be a more favorable solution as opposed to trying to further minimize latency if doing so were to introduce more packet bursts.

Additionally, the perception of the difficulty in interacting with collaborators did not have as large a decline as the perception of lag, but this trend is not as evident in the packet burst scenarios. Consistent with the number of errors for increased latencies, increasing latency does not appear to inhibit actual performance in collaborative tasks.

### Analysis of role on task performance and QoE

Since one of the users hosts the simulation server, the roles are asymmetric in nature; the connections from the host PC are different from the connections to the client PCs. We found that asymmetric roles impacted task performance but not QoE. While all three roles gave similar responses to the QoE questions, it was observed that both the users at the client computers (P2 and P3) dropped the extinguisher, on average, significantly more than the user at the server computer (P1). The number of drops, particularly with more packet bursts, was significantly different for P2 and P3 compared to P1.

Although P1 is also affected by the network conditions that P2 and P3 encounter, interactions between P2 and P3 are worsened by the added connections. For example, P2 must send data to P1, and then P1 must send packets to P3. Therefore, the network conditions can be worse since the packet bursts have a cumulative effect over the multiple connections. In a dedicated server setup, however, the roles would be effectively symmetric, at the cost of adding an additional network connection between one of the user’s computers.

### Analysis of network perspective by position

The authority model has some drawbacks in terms of presenting a consistent state. State consistency becomes a problem in any multiplayer environment that relies on users independently calculating part of the simulator state. From a user’s perspective, latency may not be apparent between some of the users. For instance, from P2’s perspective of P3 tossing the fire extinguisher to P1 may be that P1 is not experiencing latency with respect to P3. However, this is not the case; the latency is merely masked because P2 is receiving P1’s (i.e., the server’s) view of the current scene.

**Figure 8:**
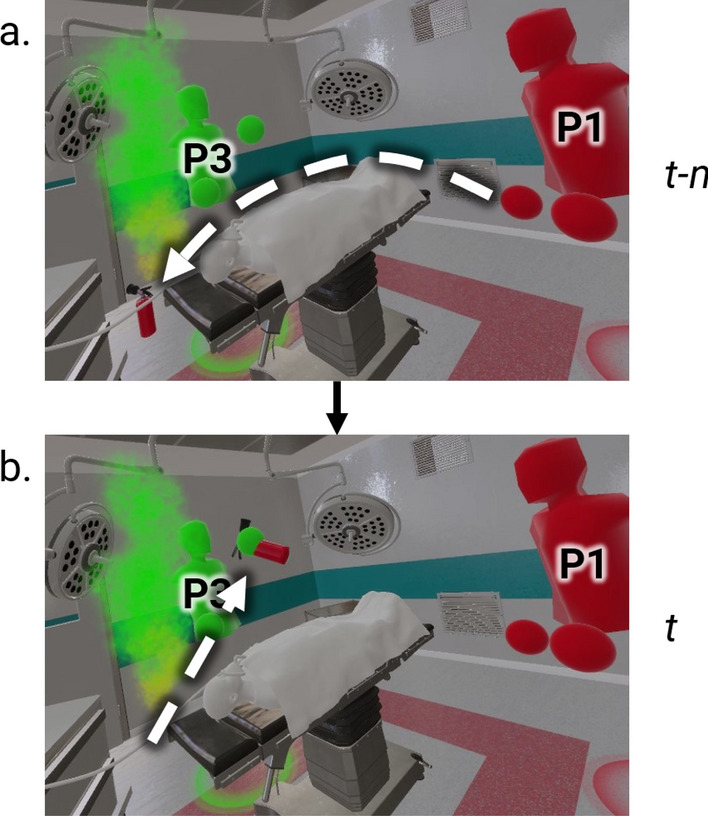
(**a**) P3 (left) appears to drop the extinguisher thrown from P1 (right) from P2’s perspective. (**b**) P3 actually catches it, but the latency between P3 and P1 gives an incorrect series of events.

Other interactions more clearly show latency. For example, P1 tossing the extinguisher to P3 will appear to have latency from P2’s perspective. This occurs because P1 is merely acting as a pass-through point for the fire extinguisher, and P3’s acquisition of authority of the fire extinguisher will not be updated by the server until after the latency period (e.g., 500ms) has passed. The latency creates a visual artifact for this example (Fig. 8); it is common to see the extinguisher pass through the receiving user and fall to the ground (Fig. 8a) only to be caught by the player later and teleport into the player’s hand (Fig. 8b). This presents an unusual phenomenon where the virtual world becomes inconsistent across multiple users and is subsequently corrected.

## Conclusion

Immersive collaborative VR applications have the potential to become the future of team training applications. However, poor quality of service can inhibit the formation of shared mental models and worsen individual and team performance, limiting dissemination to people and societies without access to reliable or high-speed internet. In this work, we show that (1) latency decreases QoE but not task performance, (2) packet bursts affect task performance and QoE more than large latency, and (3) network role significantly impacts task performance.

Since latency seems to have little effect on performance in our designed task, similar future applications should be able to exploit the finding that latency has little impact on performance, while packet bursts can have a large impact. While Vlahovic et al. found latency values as low as 200 ms can affect performance in networked PvE cooperative VR games^18^, we found users can tolerate at least 1000 ms of latency with an authority model. On the other hand, our results are consistent with the range of tolerable latencies that were introduced for omnipresent games (1000 ms)^3^. While the application presented in this paper is not omnipresent, the types of user interactions are more resilient to latency. Buffering multiple frames and then interpolating them can reduce the effects of more sensitive conditions such as high packet loss, packet coalescing, and packet bursts, and provide better user performance at the cost of adding additional latency.

There exist multiple limitations in our study that we look to address in future work. The first was the combination of latency and packet burst values. While it would be ideal for testing each of the three distinct latency values against each of the three packet burst values for each of the three distinct positions, to do so would have required a group commitment too long to request of participants. Instead, we based the testing of throttle values at 500 ms as an upper threshold to see the impact that it would have on QoE and QoS. The success of the experiments performed in this study, while rudimentary, provides baseline results to compare against subsequent, more complex studies. Future studies should investigate the effect that occurred at those other combinations or looking at a finer scale. Future research will also explore the impact that latency and packet bursts have on more complex interactions in VR, such as soft-body and fluid simulation, as those may have profound effects on the ability of someone to interact within a virtual environment.

Furthermore, while asymmetric connections do not show a significant difference in the perception of network conditions, it can penalize participants who are not hosting the game session. Depending on the application and evaluation criteria, performance can vary significantly across users in different networking roles (Video 1).

## Supplementary Information

Supplementary Information.

Supplementary Video.

## Data Availability

The data used in this study is publicly available^48^.
